# Brazilian consensus- and evidence-based recommendations in the diagnosis and treatment of pancreatic exocrine insufficiency in patients after digestive surgeries. Position paper of six brazilian medical societies of surgery

**DOI:** 10.1590/0102-67202025000042e1911

**Published:** 2025-11-28

**Authors:** Andre Luis MONTAGNINI, Wanderley Marques BERNARDO, Paulo KASSAB, Claudemiro QUIREZE, Cassio Virgílio Cavalcante de OLIVEIRA, Alessandro Landskron DINIZ, Rodrigo Nascimento PINHEIRO, Alexandre Ferreira OLIVEIRA, Pedro PORTARI, Guilherme de Andrade Gagheggi RAVANINI, Nora Manoukian FORONES, Marcus Fernando Kodama PERTILLE, Antonio Carlos VALEZI, Anna Carolina Batista DANTAS, Maira Andrade Nacimbem MARZINOTTO, Estela Regina FIGUEIRA, José JUKEMURA, Ulysses RIBEIRO, Paulo HERMAN

**Affiliations:** 1 Universidade de São Paulo, Faculty of Medicine, Department of Gastroenterology and Nutrition - São Paulo (SP), Brazil.; 2 Universidade de São Paulo, Center for Medical Education - São Paulo (SP), Brazil.; 3 Santa Casa de São Paulo, School of Medical Sciences - São Paulo (SP), Brazil.; 4 Universidade Federal de Goiás - Goiânia (GO), Brazil.; 5 Universidade Federal da Paraíba - João Pessoa (PB), Brazil.; 6 A.C. Camargo Cancer Center - São Paulo (SP), Brazil.; 7 Hospital de Base do Distrito Federal, Department of Oncologic Surgery - Brasília (DF), Brazil.; 8 Universidade Federal de Juiz de Fora - Belo Horizonte (MG) Brazil.; 9 Universidade Federal do Estado do Rio de Janeiro - Rio de Janeiro (RJ), Brazil.; 10 Escola Paulista de Medicina, Universidade Federal de São Paulo, SP, Brazil.; 11 Universidade Estadual de Londrina - Londrina (PR), Brazil.

**Keywords:** Exocrine pancreatic insufficiency, Surgery, Pancreatectomy, Esophagectomy, Gastrectomy, Bariatric surgery, Consensus, Insuficiência pancreática exócrina, Cirurgia, Pancreatectomia, Esofagectomia, Gastrectomia, Cirurgia bariátrica, Consenso

## Abstract

**Background:**

Exocrine pancreatic insufficiency (EPI) is a condition characterized by reduced exocrine secretion, leading to decreased food digestion, and digestive tract surgeries can be a cause. Postoperative “de novo” EPI is defined as the onset of digestive symptoms following surgeries, which show significant improvement after the initiation of pancreatic enzyme replacement therapy (PERT). The diagnosis of postoperative EPI may be delayed due to mild or nonspecific symptoms, both in pancreatic surgeries and in upper abdominal surgeries.

**Aims:**

The aim of this study was to conduct a systematic review on the diagnosis and treatment of “de novo” EPI related to digestive surgeries, in collaboration with the development of a consensus among the main surgical societies in Brazil.

**Methods:**

The steering committee developed 10 questions related to two areas of interest: diagnosis and treatment. A systematic review was conducted for each of the domains. The evidence was assessed for quality using the GRADEpro tool. Recommendations were formulated for each of the questions. The final report was reviewed by representatives of the surgical societies for the consolidation and approval of the recommendations through a modified Delphi system.

**Results:**

“De novo” EPI should be considered in case of the onset of postoperative digestive symptoms. Diagnostic methods vary in complexity of execution, with varying sensitivity and specificity in the postoperative condition. Fecal Elastase-1 (FE-1) has limited value in diagnosing EPI in the postoperative setting. PERT can be initiated based on clinical suspicion, and there is no difference in approach regarding the type of surgery performed. PERT should be started at the appropriate dose for the intensity of symptoms and adjusted up or down according to symptom control. Proper treatment of EPI leads to symptom improvement and an increase in quality of life. PERT should be maintained as long as patients have a favorable clinical response.

**Conclusions:**

The recommendations encompass the diagnosis and treatment of “de novo” EPI and can serve as a basis for the establishment of educational programs led by the participating surgical societies.

## ARTICLE HIGHLIGHTS

• Biliopancreatic and upper gastrointestinal surgeries are risk factors for the development of exocrine pancreatic insufficiency (EPI).

• The diagnosis of EPI is based on clinical suspicion, the presence of digestive symptoms, and laboratory tests. Active investigation of the diagnosis allows for early initiation of pancreatic enzyme replacement therapy (PERT) and reduces the risk of long-term complications.

• The first Brazilian consensus among surgical societies related to EPI led to the development of recommendations for its diagnosis, therapeutic planning, and long-term follow-up.

## Summary

**CENTRAL MESSAGE:**

Exocrine pancreatic insufficiency (EPI) is a condition in which the pancreas does not produce sufficient digestive enzymes for the proper digestion and absorption of nutrients. When left untreated, it can lead to steatorrhea, nutrient loss, chronic malnutrition, osteoporosis, and long-term impairment of quality of life. The most common cause is chronic pancreatitis, resulting from factors such as alcoholism, genetic alterations, cystic fibrosis, or obstructive conditions of the pancreas, including pancreatic tumors. Gastrointestinal surgeries may lead to the development of EPI in previously healthy patients, a condition known as “de novo” EPI. After partial or total pancreatectomy, EPI can occur in 15-100% of patients. Among upper gastrointestinal surgeries, “de novo” EPI occurs in 16-60% of cases after esophagectomy, 9-48% after bariatric surgery, and 30-100% after gastrectomy. In these cases, the cause is related to the loss of integrity of the esophagus-gastro-duodenal axis, with suppression of hormonal stimuli (cholecystokinin and secretin) and postprandial gastrointestinal asynchrony.

**PERSPECTIVES:**

Pancreatic and upper gastrointestinal surgeries are significant risk factors for “de novo” EPI or aggravate it if already present. Subclinical manifestations, nonspecific symptoms, and lack of awareness may delay EPI diagnosis, leading to nutritional impairment and reduced quality of life. The role of this first Brazilian consensus, validated by national surgical societies, is to serve as a foundation for the development of educational and training programs for surgeons.

## RESUMO

**Racional:**

A insuficiência pancreática exócrina (IPE) é a situação em que ocorre redução da secreção exócrina e consequente diminuição da digestão alimentar e as cirurgias do trato digestivo podem ser sua causa. A IPE “de novo” é definida como o aparecimento de sintomas de digestivos após cirurgias e que apresentam melhora significativa com a instituição da terapia de reposição de enzimas pancreáticas (TREP). O diagnóstico da IPE “de novo” pode ser tardio em virtude dos sintomas serem leves ou pouco específicos, tanto nas cirurgias pancreáticas quanto nas cirurgias abdominais superiores.

**Objetivos:**

Revisão sistemática sobre diagnóstico e tratamento sobre a IPE “de novo” ligada às cirurgias digestivas com a colaboração e desenvolvimento de um consenso com as principais sociedades de cirurgia do Brasil.

**Métodos:**

O comitê diretivo elaborou 10 questões relacionadas a dois domínios de interesse: diagnóstico e tratamento. Foram realizadas buscas sistemáticas para cada um dos domínios. As evidências foram avaliadas em relação à qualidade pelo instrumento GRADEpro. Para a cada uma das questões foram elaboradas recomendações. O relatório final foi avaliado pelos representantes das sociedades de cirurgia para a consolidação e aprovação das recomendações pelo sistema Delphi modificado.

**Resultados:**

A IPE “de novo” deve ser considerada quando houver aparecimento de sintomas digestivos pós-operatórios. Os métodos diagnósticos apresentam variados graus de complexidade na execução com sensibilidade e especificidade variadas na condição de pós-operatório. A Elastase-1 fecal (FE-1) tem pouco valor no diagnóstico de IPE no pós-operatório. A TREP pode ser iniciada com base na suspeita clínica e não existe diferença na abordagem em relação ao tipo cirurgia realizada. Iniciar a TREP com a dose adequada para a intensidade dos sintomas e ajustada para mais ou menos de acordo com a resposta clínico. O correto tratamento da IPE proporciona melhora dos sintomas e aumento na qualidade de vida. A TREP deve ser mantida enquanto houver resposta clínica.

**Conclusões:**

As recomendações deste consenso abrangem o diagnóstico e tratamento da IPE “de novo” e podem servir de base para a instituição de programas de educação, capitaneados pelas sociedades cirúrgicas participantes.

## INTRODUCTION

Exocrine pancreatic insufficiency (EPI) is a condition characterized by insufficient production of digestive enzymes by the pancreas, resulting in impaired digestion and absorption of dietary nutrients^57^. When left untreated, it can cause steatorrhea, nutrient loss, chronic malnutrition, osteoporosis, and long-term reduction in quality of life (QOL). The most common cause is chronic pancreatitis, which may arise from factors such as alcoholism, genetic mutations, cystic fibrosis, or pancreatic obstruction, including tumors. Management of EPI involves oral pancreatic enzyme replacement therapy (PERT), with dosage tailored to the degree of insufficiency in each patient^79^.

Surgeries of the digestive tract can lead to the development of EPI in previously unaffected individuals, a condition referred to as “de novo” EPI^26^. In partial or total pancreatectomy, EPI occurs in 15-100% of cases, depending on the extent of resection, the functional capacity of the remaining pancreas, and the potential loss of the duodenum, which is essential for stimulating pancreatic secretion and activating digestive enzymes^35^. Among upper gastrointestinal procedures, “de novo” EPI develops in 16-60% of patients following esophagectomy^3^, in 9-48% after bariatric surgery^15^ ^,^ ^38^, and in 30-100% after gastrectomy. In these cases, the underlying mechanism is associated with disruption of the esophagogastroduodenal axis, leading to suppression of hormonal stimuli (cholecystokinin and secretin) and postprandial gastrointestinal asynchrony^47^. If untreated, EPI can result in digestive manifestations such as malabsorption, steatorrhea, vitamin deficiencies, and malnutrition^17^. For this reason, it is essential that surgeons remain vigilant for the development of EPI in patients with previously normal exocrine function who have undergone upper gastrointestinal surgery. Early recognition and treatment of EPI are critical to preventing malnutrition in this population.

## OBJECTIVES

Given the importance of early recognition of EPI and the need to mitigate its adverse effects in patients undergoing digestive surgery, this systematic review was conducted to evaluate the diagnosis and treatment of postoperative EPI and to develop evidence-based recommendations.

### Participating medical societies

The Division of Digestive System Surgery of the Department of Gastroenterology and Nutrition at the School of Medicine, Universidade de São Paulo, coordinated this systematic review. Notably, two representatives with expertise in the area were formally invited from each of the following Brazilian surgical societies: Colégio Brasileiro de Cirurgia Digestiva, Colégio Brasileiro de Cirurgia Hepato-Biliopancreática, Sociedade Brasileira de Cirurgia Oncológica, Colégio Brasileiro de Cirurgia, Associação Brasileira do Câncer Gástrico e Sociedade Brasileira de Cirurgia Bariátrica e Metabólica.

## METHODS

### Collaboration groups

In total, three working groups were established:

1. Formulation of research questions,
2. Identification of evidence through systematic review, and
3. Development of consensus-based recommendations.

### Preparation of questions

With regard to postoperative EPI, the group identified two domains of interest comprising a total of 10 questions:

1. Diagnostic domain:
  • When should postoperative EPI be clinically suspected?
  • Which diagnostic tests can be applied?
  • Is fecal elastase testing mandatory for the diagnosis of postoperative EPI?
2. Treatment domain (PERT):
  • Does it depend on clinical evaluation?
  • Does it depend on laboratory tests?
  • Is there any difference in enzyme replacement therapy strategy according to the type of surgery (pancreatectomy, esophagectomy, gastrectomy, bariatric surgery)?
  • What is the dosing strategy (fixed dose, increasing dose, decreasing dose)?
  • How should the response to replacement therapy be assessed?
  • What is the impact of pancreatic enzyme replacement therapy on clinical outcomes/quality of life/long-term survival?
  • For how long should PERT be maintained?

### Eligibility criteria for the evidence to be included (PICO)

• Patients: with suspected or confirmed diagnosis of exocrine pancreatic insufficiency (primary or secondary);
• Intervention or Exposure: undergoing a diagnostic method or enzyme replacement therapy;
• Control: not undergoing the diagnostic method or treatment;
• Outcomes: diagnostic accuracy or clinical outcomes of efficacy and harm.

Included study designs: comparative observational studies (cohort or cross-sectional) and experimental studies (randomized or nonrandomized clinical trials). No time or language restrictions. Full text available or abstract with data of interest.

### Sources of scientific information consulted and search strategies

Searches were conducted in the Medline and Embase databases, supplemented by a manual review of the references of selected articles, as well as complementary searches in Google Scholar and ClinicalTrials.gov.

The search strategies used for the Medline and Embase databases were similar, differing only in the complementary searches.

Medline/Embase:

#1 (“Exocrine Pancreatic Insufficiency”[All Fields] OR “Exocrine Pancreatic Insufficiencies”[All Fields] OR “Pancreatic Insufficiency”[All Fields] OR “Pancreatic Insufficiencies”[All Fields] OR (“Exocrine Pancreatic Insufficiency”[MeSH Terms] OR (“exocrine”[All Fields] AND “pancreatic”[All Fields] AND “insufficiency”[All Fields]) OR “Exocrine Pancreatic Insufficiency”[All Fields] OR (“Exocrine Pancreatic Insufficiency”[MeSH Terms] OR (“exocrine”[All Fields] AND “pancreatic”[All Fields] AND “insufficiency”[All Fields]) OR “Exocrine Pancreatic Insufficiency”[All Fields] OR (“exocrine”[All Fields] AND “pancreatic”[All Fields] AND “insufficiencies”[All Fields]) OR “Exocrine Pancreatic Insufficiencies”[All Fields]) OR (“Exocrine Pancreatic Insufficiency”[MeSH Terms] OR (“exocrine”[All Fields] AND “pancreatic”[All Fields] AND “insufficiency”[All Fields]) OR “Exocrine Pancreatic Insufficiency”[All Fields] OR (“pancreatic”[All Fields] AND “insufficiency”[All Fields]) OR “Pancreatic Insufficiency”[All Fields]) OR (“Exocrine Pancreatic Insufficiency”[MeSH Terms] OR (“exocrine”[All Fields] AND “pancreatic”[All Fields] AND “insufficiency”[All Fields]) OR “Exocrine Pancreatic Insufficiency”[All Fields] OR (“pancreatic”[All Fields] AND “insufficiencies”[All Fields]) OR “Pancreatic Insufficiencies”[All Fields])))

#2 ((sensitiv*[Title/Abstract] OR sensitivity and specificity[MeSH Terms] OR diagnose[Title/Abstract] OR diagnosed[Title/Abstract] OR diagnoses[Title/Abstract] OR diagnosing[Title/Abstract] OR diagnosis[Title/Abstract] OR diagnostic[Title/Abstract] OR diagnosis[MeSH:noexp] OR (diagnostic equipment[MeSH:noexp] OR diagnostic errors[MeSH:noexp] OR diagnostic imaging[MeSH:noexp] OR diagnostic services[MeSH:noexp]) OR diagnosis, differential[MeSH:noexp] OR diagnosis[Subheading:noexp]))

#3 (((clinical[Title/Abstract] AND trial[Title/Abstract]) OR clinical trials as topic[MeSH Terms] OR clinical trial[Publication Type] OR random*[Title/Abstract] OR random allocation[MeSH Terms] OR therapeutic use[MeSH Subheading]) OR Comparative study OR Comparative studies OR Epidemiologic methods)

#4 ((“Exocrine Pancreatic Insufficiency” OR “Exocrine Pancreatic Insufficiencies” OR “Pancreatic Insufficiency” OR “Pancreatic Insufficiencies”)) OR ((Exocrine Pancreatic Insufficiency OR Exocrine Pancreatic Insufficiencies OR Pancreatic Insufficiency OR Pancreatic Insufficiencies))

#5 ((#1 AND #2) OR (#1 AND #3)) OR #4 = 6.650 (Medline) + 76 (Embase)

Supplementary search: Google Scholar and ClinicalTrials.gov:

#1 “Exocrine Pancreatic Insufficiency” AND random* #2 “Exocrine Pancreatic Insufficiency” AND diagnosis #3 (#1 OR #2)

### Data extraction and collection

Extracted data included the first author’s name, year of publication, patient characteristics, diagnostic methods and reference standards, treatment approaches and comparators, clinical outcomes related to efficacy and risk, and duration of follow-up.

### Assessment of risk of bias and quality of evidence

Cross-sectional diagnostic studies were evaluated using the QUADAS-2^81^ instrument, which considers the following domains: patient selection, index test, reference standard, flow, and timing. Observational comparative studies were assessed with the Robins I^71^ instrument, addressing selection, confounding, classification of interventions, measurement of exposures, missing data, outcome assessment, and reporting of results.

The risk of bias in randomized clinical trials was assessed using the RoB II^72^ ^,^ ^73^ instrument based on the following criteria: randomization process, allocation concealment, double-blinding, blinding of outcome assessors, losses to follow-up, outcome measurement, prognostic characteristics, use of intention-to-treat analysis, sample size calculation, and early trial discontinuation. The overall risk of bias was classified as very low, low, or high.

When outcome analyses were not aggregated through meta-analysis, the quality of evidence was inferred directly from the assessed risk of bias. For outcomes analyzed via meta-analysis, the quality of evidence was evaluated using the GRADEpro^24^instrument, taking into account study design (observational cohort or randomized trial), risk of bias, inconsistency, imprecision, indirectness, and publication bias. In this context, the quality of evidence was rated as very low, low, moderate, or high.

### Expression of results and analysis

Diagnostic accuracy results were expressed as sensitivity and specificity with 95% confidence intervals. Clinical outcomes were reported as absolute risk for comparisons, as risk differences for categorical variables, and as mean differences with standard deviations and 95% confidence intervals for continuous variables.

### Consensus

Consensus was reached using the modified Delphi methodology^65^, where agreement was defined as at least 80% among evaluators. All recommendations were submitted to the evaluation group. Recommendations that did not achieve consensus in the first round were revised and subjected to further review.

## RESULTS

**Figure 1.**
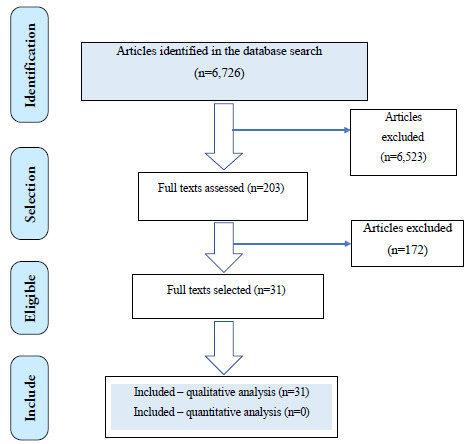
Diagram of evidence search and article selection: EPI DIAGNOSIS.

**Figure 2.**
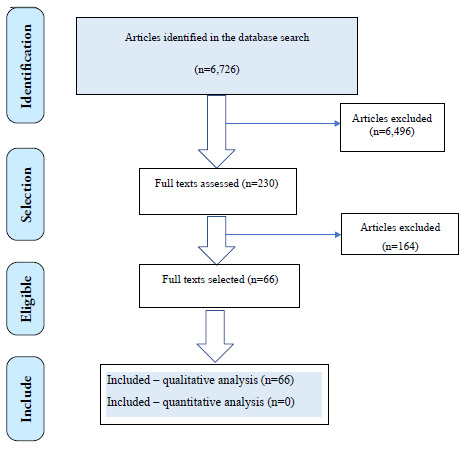
Diagram of evidence search and article selection: EPI TREATMENT.

A total of 6,726 records were retrieved from the Medline and Embase databases. Based on the eligibility criteria, 203 articles were selected for full-text review addressing diagnostic questions (Figure 1), and 230 articles were selected for treatment-related questions (Figure 2).

In total, 31 studies were selected to inform the evidence synthesis for the three clinical diagnostic questions. The overall quality of evidence was considered low, with limiting factors including the absence of a reference standard, non-consecutive case series, and lack of independence and blinding in comparative assessments.

On the whole, 66 studies were selected to support the evidence synthesis for the seven clinical treatment questions. The overall quality of evidence was considered very low, with the main limitations being confounding bias, selection bias, and reported bias.

All recommendations achieved >80% agreement in the first round of Delphi voting, and no additional rounds were required.

### Diagnostic domain

#1.1 When should postoperative EPI be clinically suspected?

**Table:** 

**Evidence summary** Initial suspicion of EPI is based on the appearance of abdominal symptoms such as distension, flatulence, increased bowel movements, soft stools, weight loss, and vitamin deficiencies (A, D, E, and K). In cases of mild symptoms, confusion with other gastrointestinal disorders may occur. Laboratory studies or a therapeutic trial may be necessary. **Quality of evidence:** LOW **Recommendation:** Monitor patients in the postoperative follow-up and identify possible signs and symptoms suggestive of EPI. **CONSENSUS:** 100%

Typical manifestations of EPI include steatorrhea, decreased stool consistency, increased bowel movements, foul-smelling stools, flatulence, weight loss, and loss of muscle mass^28^ ^,^ ^33^ ^,^ ^37^. Patients undergoing pancreatic resection develop some degree of clinical manifestations in up to 70% of cases, with increased bowel movements and abdominal distension being the most common^64^ ^,^ ^77^. De novo EPI is defined as the development of symptoms (steatorrhea, abdominal distension, abdominal cramps, and weight loss) postoperatively, with clinical improvement following initiation of PERT^3^. Postoperative clinical manifestations correspond with quantitative stool fat tests, typically appear when pancreatic lipase and trypsin secretion decline to less than 10% of normal, and are associated with low levels of fat-soluble vitamins (A, D, E, and K)^1^ ^,^ ^6^ ^,^ ^42^ ^,^ ^43^. In surgically treated patients who develop EPI, PERT results in significant symptom relief and weight gain^35^ ^,^ ^56^. The clinical manifestations of EPI can range from mild to severe^39^. Steatorrhea may be subclinical when patients restrict dietary fat intake to minimize symptoms, and diarrhea may not occur. Symptoms of mild EPI include abdominal distension and cramping, which can be easily overlooked or misattributed in the absence of clinical suspicion^30^. Symptom severity is influenced by pancreatic enzyme secretion, dietary fat intake, and intestinal transit time^13^ ^,^ ^46^ ^,^ ^75^. The symptoms of EPI are nonspecific and primarily gastrointestinal. In clinical practice, initial suspicion is based on patient evaluation and reports of changes in bowel habits and weight loss. However, relying solely on patient-reported symptoms can lead to both underdiagnosis and overdiagnosis^52^. In such cases, initiation of PERT with subsequent clinical improvement can confirm the diagnosis^62^.

#1.2 Which diagnostic tests can be applied?

**Table:** 

**Evidence summary** The main diagnostic tests for exocrine pancreatic insufficiency are the fecal elastase test (FE-1), the qualitative fecal fat test, and the modified 13C-mixed triglyceride breath test (13C-MTGT). They present variable sensitivity and specificity and may be affected by the patient’s clinical conditions. The remaining pancreatic volume can predict EPI after pancreatic resection, and non-alcoholic fatty liver disease resulting from EPI can be assessed by abdominal CT. **Quality of evidence:** LOW **Recommendation:** Considering the difficulties in performing and the costs of more complex tests, their use should be limited to specific cases. **CONSENSUS:** 100%

There are direct and indirect methods for assessing EPI. Direct measurements typically require duodenal cannulation and aspiration of pancreatic secretions following intravenous administration of secretagogues. Although highly sensitive and specific, direct methods are invasive, time-consuming, costly, and have limited applicability in routine clinical practice^75^. Indirect tests, in contrast, assess pancreatic enzyme activity or byproducts of enzymatic digestion.

Fecal absorption coefficient/coefficient of fat absorption (CFA): This test requires a 72-hour stool collection to quantify fecal fat and is considered the “gold standard” among indirect tests for estimating EPI. Patients must consume a high-fat diet and refrain from using PERT during the study. Its clinical application is limited due to labor intensity and high demands on laboratory staff.

Fecal Elastase-1: Measures elastase-1 concentration in a 1-3 g stool sample. Normal levels are >200 μg/g; moderate insufficiency is 100-200 μg/g, and severe insufficiency is <100 μg/g. This test is widely used in clinical practice, though results may be affected by liquid stool and the amount of remaining pancreatic tissue^46^ ^,^ ^75^.

Qualitative Fecal Fat Test: Detects fecal fatty acids and neutral fats in a single 3-g stool sample. Patients should consume >60 g of fat daily for at least 3 days. Normal total fat concentration is <100 drops/hpf, and normal neutral fat concentration is <60 drops/hpf.

Modified ^13^C-Mixed Triglyceride Breath Test (^13^C-MTGT): This test noninvasively and reliably identifies moderate EPI, but requires prolonged breath sampling (6 h). The preferred parameter for assessing pancreatic exocrine function, as reported by several groups, is the cumulative exhalation of ^13^CO_2_ (% of the administered dose) over 5-8 h. This measurement correlates well with pancreatic lipase secretion; however, the long sampling period and requirement for patient immobilization limit its routine clinical application^13^ ^,^ ^30^ ^,^ ^46^ ^,^ ^75^. Additional indicators of EPI or risk of its development include the presence of non-alcoholic fatty liver disease (NAFLD) in patients undergoing pancreatic surgery^33^ as well as imaging assessments of the pancreatic remnant (<24.5 mL) or residual thickness (<11.4 mm) on computed tomography, which may suggest existing EPI or an increased risk of its development^29^ ^,^ ^53^.

# 1.3 Is fecal elastase testing mandatory for the diagnosis of postoperative EPI?

**Table:** 

**Evidence summary** In patients undergoing pancreatectomy, gastrectomy, and esophagectomy, FE-1 determination has limited value as a definitive diagnostic test for EPI. FE-1 results should not be used in isolation. **Quality of evidence:** LOW **Recommendation:** If laboratory documentation of EPI is required, the test may be requested in addition to the clinical assessment; however, the results should be interpreted with caution. **CONSENSUS:** 93%

In non-operated patients, the FE-1 threshold below which steatorrhea should be suspected is very low, at 15 μg/g. Following pancreatic resection, this threshold increases substantially to 207 μg/g, reducing its usefulness for predicting steatorrhea in operated patients^2^. In a study of 40 patients undergoing pancreatectomy, using an FE-1 cutoff of 200 μg/g for diagnosing EPI versus CAF yielded a diagnostic accuracy of 70%, with a sensitivity (95%CI) of 91% (83-96%), specificity (95%CI) of 35% (22-51%), positive predictive value of 70%, and negative predictive value of 71%. These findings suggest that the optimal FE-1 cutoff for diagnosing EPI, defined by a CFA <93%, is less than 128 μg/g. Using this threshold, the area under the ROC curve (AUC) was 0.71 (p=0.0001), with a sensitivity (95%CI) of 90% (82-96%), specificity (95%CI) of 44% (30-60%), positive predictive value of 75%, and negative predictive value of 71%. Overall, even when diagnostic thresholds are optimized via ROC curve analysis, FE-1 performs poorly against CAF in diagnosing EPI, with specificity never exceeding 50%^27^. Similarly, in patients undergoing esophagectomy and gastrectomy, particularly total or partial procedures with Roux-en-Y reconstruction, the diagnostic accuracy of FE-1 for EPI is very low, and it should not be used as a standard assessment tool^10^ ^,^ ^19^ ^,^ ^62^.

### Treatment domain

# 2.1 Does the initiation of PERT depend on clinical evaluation?

**Table:** 

**Evidence summary** In patients undergoing pancreatectomy, esophagectomy, gastrectomy, and bariatric surgery, clinical manifestations (flatulence, dyspeptic symptoms, abdominal distension, diarrhea, and steatorrhea) and those associated with nutrient loss (particularly weight loss and fat-soluble vitamin deficiency) are strongly associated with EPI. **Quality of evidence:** VERY LOW **Recommendation:** For patients undergoing total pancreatectomy, PERT should be initiated immediately postoperatively. In other cases, PERT should be guided by clinical suspicion. **CONSENSUS:** 93%

In this study, de novo EPI was defined as the development of symptoms (steatorrhea, abdominal distension, abdominal cramps, and weight loss) after resection, with subsequent initiation of pancreatic enzyme replacement therapy leading to symptom resolution^26^ ^,^ ^64^. EPI becomes clinically apparent when pancreatic lipase levels are markedly reduced, often accompanied by multiple markers of malnutrition, including weight loss, vitamin deficiencies, electrolyte imbalance, osteoporosis, and osteomalacia, resulting in bone fractures^13^. Postoperative EPI may be subclinical or manifest with symptoms secondary to undigested food in the intestinal lumen (fatty diarrhea, flatulence, and dyspeptic symptoms) and/or those associated with nutrient loss (weight loss, fat-soluble vitamin deficiencies)^62^. Steatorrhea may be occasional after fatty meals or present as frequent episodes^34^. The prevalence of EPI after pancreatectomy varies by type of surgical resection: lower in partial pancreatectomies, higher in extended pancreatectomies and pancreaticoduodenectomies, and universally present in total pancreatectomies^46^. Following esophagectomy, EPI can occur in up to 57% of patients^3^, and after partial gastrectomy, in up to 60%, particularly in those undergoing Roux-en-Y reconstruction^47^. EPI may also occur after bariatric surgery^38^. When assessed using the ^13^C-MTGT, EPI was present in >70% of cases and correlated with the type of procedure: low prevalence in gastric sleeve (4%), intermediate in Roux-en-Y gastric bypass (RYGB) (8.3%), and exceeding 70% in biliopancreatic diversion with duodenal switch^78^.

# 2.2 Does the initiation of PERT depend on laboratory tests?

**Table:** 

**Evidence summary** Diagnostic tests for EPI have low accuracy and should be used in conjunction with clinical evaluation, particularly when indicating pancreatic enzyme supplementation therapy. This approach helps avoid underdiagnosis, which could delay appropriate treatment for postoperative patients with suspected or at-risk EPI. **Quality of evidence:** VERY LOW **Recommendation:** Consider the response in 2.1. **CONSENSUS:** 93%

EPI during the first postoperative year was defined as the need for PERT within 1 year after surgery and/or an abnormal pancreatic exocrine function test. PERT was initiated primarily based on clinical indications, and in most patients, abnormal function tests confirmed the need for therapy. Accuracy rates for clinical symptoms, including steatorrhea, were 62% for fecal testing and 88% for breath testing^27^ ^,^ ^61^. In a study of 40 patients undergoing cancer surgery, FE-1 was compared to CAF using a cutoff of 200 mcg/g for EPI. The diagnostic accuracy of FE-1 was 70%, with a sensitivity of 91%, specificity of 35%, positive predictive value of 70%, and negative predictive value of 71%. No clear association was observed between CAF levels and FE-1. The sensitivity and specificity of FE-1 for detecting steatorrhea in surgical patients were as follows: FE-1<200 mcg/g, 100% and 83.3%; FE-1<100 mcg/g, 100% and 100%; and FE-1<15 mcg/g, 61.8 and 100%, respectively. EPI can be assumed in patients with symptoms suggestive of malabsorption; however, the absence of clinical steatorrhea is not sufficient to exclude the diagnosis. Thus, pancreatic function tests may be useful in identifying EPI in asymptomatic patients^75^. Assessment of pancreatic remnant thickness and volume via CT may provide prognostic information regarding the development of EPI. A pancreatic tail thickness less than 11.4 mm predicts abnormal ^13^C-MTGT results after resection, with a sensitivity of 88.9% and specificity of 70%^29^ ^,^ ^48^. Remnant pancreatic volume below 24.1 mL was identified as the only independent predictive factor for postoperative EPI (p<0.001; hazard ratio: 5.94, 95% confidence interval: 2.96-12.3)^53^.

Postoperative EPI, assessed using ^13^C-MTGT or FE-1, has been diagnosed in 68-74%^30^ ^,^ ^39^ of patients following pancreatectomy. After pancreatic resection, FE-1 has limited utility in predicting steatorrhea^2^ and shows poor correlation with CAF^20^ ^,^ ^27^. Therefore, FE-1 should not be used as the sole diagnostic tool for EPI following pancreatoduodenectomy (PD)^11^.

# 2.3 Is there any difference in enzyme replacement strategy according to the type of surgery (pancreatectomy, esophagectomy, gastrectomy, bariatric surgery)?

**Table:** 

**Evidence summary** The risk of EPI is individual to each patient; therefore, pancreatic enzyme replacement therapy should take into account the clinical manifestations and diagnostic tests of each patient. However, this risk may vary depending on multiple factors, such as the remaining volume of the pancreas, the type of pancreatic resection technique, or esophagogastric surgeries. **Quality of evidence:** VERY LOW **Recommendations:** Clinical and anatomical variables of each patient should be considered to establish individual risk for the development of EPI. Once EPI is diagnosed, PERT should be initiated regardless of the type of surgery performed. **CONSENSUS:** 100%

The type of pancreatectomy significantly influences the development of de novo EPI; greater pancreatic resection is associated with a higher risk^30^. Several studies indicate that PD has a higher incidence than distal pancreatectomy (DP)^23^ ^,^ ^40^, whereas central pancreatectomy (CP) is associated with the lowest incidence among pancreatic resections^41^ ^,^ ^58^. The remaining pancreatic volume, assessed by volumetry, and the thickness of the distal pancreas are correlated with the development of EPI^30^. Following PD, the prevalence of EPI may increase over time, rising from 8% to 20% after 29 months of follow-up. In such cases, there is a greater need for PERT, with doses adjusted according to clinical worsening^14^ ^,^ ^23^. When evaluated solely by FE-1, one study reported no significant decrease over 24 months, suggesting a poor correlation with clinical status^68^. The modality of pancreatic stump reconstruction (pancreato-gastric anastomosis>pancreato-jejunal anastomosis), remnant pancreatic texture (hard>soft), and the presence of pancreatic duct dilation (potential anastomotic stricture) are significant risk factors for the development of EPI^2^ ^,^ ^7^ ^,^ ^8^ ^,^ ^49^ ^,^ ^61^. In patients with pancreatic and periampullary tumors, the mean preoperative prevalence of EPI was 44% before pancreatoduodenectomy, 20% before DP, and 63% before total pancreatectomy. At least 6 months postoperatively, the prevalence of EPI increased to 74% after pancreatoduodenectomy, 67% after DP, and 100% after total pancreatectomy. These findings underscore the importance of pancreatic enzyme supplementation in this population^77^.

Upper gastrointestinal surgeries can also affect pancreatic exocrine function. In patients undergoing partial gastrectomy for cancer, pancreatic function was assessed pre- and postoperatively, comparing reconstruction modalities, B-I versus Roux-en-Y anastomosis. Patients with Roux-en-Y reconstruction exhibited significantly worse pancreatic function scores, higher diarrhea scores (PGSAS-37), and increased loose stool frequency compared with B-I patients^50^. A comparison between total gastrectomy and partial gastrectomy with Roux-en-Y reconstruction revealed that 33% of patients experienced a decrease in FE-1 values (<200 mcg/g) during the first postoperative year, although diarrhea was not observed. Surgeons should be aware of the possibility of subclinical EPI and consider initiating PERT when indicated^69^. Systematic implementation of a PERT-supplemented diet for 12 months resulted in significantly better nutritional outcomes, including prealbumin levels and Gastrointestinal Quality of Life Index (GIQLI) scores, compared with patients who did not receive PERT^9^. Following gastrectomy or esophagectomy, 66.7% of patients developed diarrhea, but only 44% had FE-1 <200 mcg/g. Over a 24-month follow-up, the prevalence of postoperative EPI increased to 73%, accompanied by decreases in vitamin levels, body weight, and lean mass. All patients with EPI developed sarcopenia between 18 and 24 months postoperatively^31^. In bariatric surgery, EPI has been increasingly recognized as a potential complication, occurring in 41.6% of patients. RYGB and biliopancreatic diversion with duodenal switch were associated with the highest incidence of EPI. PERT resolved symptoms in >80% of cases^44^. The prevalence of EPI is significantly higher in patients undergoing RYGB compared with those undergoing gastric sleeve procedures^4^ ^,^ ^12^ ^,^ ^38^.

# 2.4 What is the dosing strategy (fixed dose, increasing dose, decreasing dose)?

**Table:** 

**Evidence summary** The patient’s clinical response to PERT determines the necessary dose adjustments. **Quality of evidence:** VERY LOW **Recommendation:** In our setting, only capsules with coated microbeads in doses of 10,000 and 25,000 units of lipase are available. It is recommended to start with doses of 25,000 to 50,000 units at main meals and half of that dose at snacks. In the initial phases of PERT, frequent clinical evaluations are recommended to adjust the total daily dose. **CONSENSUS:** 100%

Once the diagnosis of EPI is established, treatment should be initiated promptly. PERT should begin at a low dose, adjusted according to symptom severity. Capsules should be taken with food, ideally 5-10 minutes before or at the start of a meal, and remain effective for up to 60 minutes. Daily lipase doses should not exceed 10,000 units/kg body weight, and doses above 6,000 units/kg per meal have been associated with fibrosing colonopathy^46^. Initial dosing typically ranges from 10,000 to 50,000 units with snacks and 50,000 to 75,000 units with main meals. Regular monitoring of clinical and laboratory response should guide dose adjustments^18^. Current formulations include acid-resistant capsules containing enzyme microspheres in a pH-sensitive polymer. For uncoated tablets, concomitant use of an H_2_ receptor blocker or proton pump inhibitor is required to control gastric pH. As it is a high-cost therapy, its use should be optimized. Patients may be advised to adjust enzyme doses (upward for larger or high-fat meals and downward for smaller or low-fat meals). Doses >120,000 lipase units per meal are rarely required^2^. In patients undergoing bariatric or esophageal surgery with a diagnosis of EPI, enzyme replacement therapy has resulted in symptom improvement in up to 85% of cases. Randomized trials comparing PERT with placebo after gastrectomy demonstrated improvements in bowel movement frequency, stool consistency, overall symptoms, prealbumin levels, and quality-of-life after 3 months, although no significant effect on weight gain was observed^25^. Following pancreatectomy, lipase dose adjustments should be based on clinical or nutritional symptoms, as no consistent association has been demonstrated between symptom severity and FE-1 levels^37^. In patients with neuroendocrine tumors, the use of somatostatin analogs after DP frequently induces EPI, and PERT appears to improve both survival and nutritional status^76^. Total pancreatectomy invariably results in absolute EPI, requiring lifelong PERT. Although no specific replacement guidelines exist for these cases, average daily enzyme consumption can reach 175,000 lipase units. Diarrhea control is achieved in approximately 88% of patients. BMI typically decreases soon after initiating therapy; however, in the long term, up to 80% of patients regain values close to preoperative BMI^21^.

# 2.5 How should the response to replacement therapy be assessed?

**Table:** 

**Evidence summary** The patient’s clinical and laboratory responses to pancreatic enzyme replacement therapy reflect its effectiveness, adverse events, and the need for treatment monitoring. **Quality of evidence:** VERY LOW **Recommendation:** Maintain patient follow-up with regular evaluations. **CONSENSUS:** 100%

In bariatric surgery, patients undergoing RYGB with fecal elastase <500 mcg/g who received PERT at a dose of 30,000 U lipase/day for 3 months showed a reduction in clinical symptoms of EPI without compromising weight loss^55^. Assessment of clinical and laboratory parameters, including vitamin D levels, is a good indicator of response to PERT. In patients who continued to experience symptoms, dose escalation led to further clinical improvement^56^. In a prospective, randomized, double-blind, placebo-controlled trial, patients undergoing PD with postoperative fecal elastase <200 μg/g were assigned to receive either 120,000 units of lipase per day (n=151) or placebo (n=153) for 3 months. PERT resulted in significantly greater weight gain and improvement in prealbumin levels compared with placebo, although no significant difference was observed in QOL scores. Poor adherence to PERT was identified as a significant risk factor for weight loss (p<0.001)^36^. Weight loss after esophagectomy remains a major challenge. In a study of patients with symptoms of EPI following esophagectomy, FE levels were measured, and patients were divided into two groups: FE-1 <200 μg/g and FE-1 200-500 μg/g. All of them received PERT. Among those with FE-1 <200 μg/g, symptom improvement occurred in 90% and weight gain in 70% of cases. In the group with FE-1 between 200 and 500 μg/g, symptom improvement was reported in 42% and weight gain in 17%^32^. In patients undergoing oncological surgery for pancreatic neoplasia, early initiation of PERT was associated with a reduction in EPI symptoms and improvement in QOL^45^.

# 2.6 What is the impact of PERT on clinical outcomes/quality of life/long-term survival?

**Table:** 

**Evidence summary** Patients who respond to PERT show symptom reduction and improved quality of life, which remains stable in follow-ups beyond 12 months. **Quality of evidence:** VERY LOW **Recommendation:** Continue PERT and guide patients on individual dose adjustments in cases of significant changes in the nutritional quality of a given meal. **CONSENSUS:** 100%

In a study of patients undergoing pancreatectomy (PD and DP) for benign or malignant diseases with a diagnosis of steatorrhea and diarrhea, PERT was administered. The number of patients with steatorrhea and diarrhea gradually decreased postoperatively, returning to preoperative levels within 12 months. Stool elastase levels declined after surgery and remained low throughout the 60-month follow-up (p=0.009). Global health status, physical functioning, role functioning, fatigue, nausea and vomiting, loss of appetite, and financial difficulties were significantly associated with uncontrolled EPI^66^ ^,^ ^67^ ^,^ ^73^. Patients undergoing PD with pancreatogastrostomy reconstruction were compared with those undergoing subtotal gastrectomy (SG) for distal gastric tumors. No differences were observed in median QOL scores (GIQLI) between the PD and SG groups. Overall, four patients in the PD group, but none in the SG group, developed steatorrhea. In addition to exocrine insufficiency, concomitant gastrectomy in the PD group was a major factor contributing to the inability to regain weight^54^. Two prospective, randomized, controlled trials evaluated the efficacy and safety of pancreatin minimicrosphere capsules containing 12,000 or 25,000 units of lipase compared with placebo for the treatment of EPI after pancreatic resection. One trial included a 1-week double-blind phase, and in one study, this was followed by an open-label extension with PERT for 1 year. In both the double-blind and open-label phases, least-squares mean changes in CAF were significantly greater with pancreatin compared with placebo, p<0.001. No adverse events led to study discontinuation, and no serious adverse events or deaths occurred during the double-blind phase^63^ ^,^ ^80^. Patients undergoing high-risk PD (HR-PD) or total pancreatectomy (TP) reported similar QOL outcomes at 30 months, as assessed by the EQ-5D-3L, EORTC QLQ-C30, and EORTC QLQ-PAN26 questionnaires. The most frequent complaints were fatigue, diarrhea, and insomnia. All patients undergoing TP required PERT, compared with 63% of patients in the HR-DP group (p<0.01), with a significantly higher number of capsules per day (13 vs. 6; p<0.01)^42^.

# 2.7 For how long should PERT be maintained?

**Table:** 

**Evidence summary** Patient adherence to PERT is a limiting factor for long-term maintenance; however, in the presence of a therapeutic response (efficacy and safety), therapy should be continued. **Quality of evidence:** VERY LOW **Recommendation:** The probability of spontaneous reversal of EPI is very low. Excluding cases of adverse events or allergies, PERT should be maintained indefinitely. Dose adjustments may be required during follow-up. **CONSENSUS:** 100%

The occurrence of EPI in surgical patients was evaluated in a prospective study of 108 individuals undergoing pancreatic resections, with 74% presenting with EPI. Among patients without preoperative symptoms suggestive of EPI, 54% developed de novo EPI postoperatively. The most frequent concern, reported by 56% of these patients, was the fear of forgetting to take medication with meals. Another group included patients who had been on PERT for more than 3 months, of whom over 60% reported no impairment related to treatment. When asked whether they would be willing to change their lifestyle to avoid taking PERT, only 4.7% answered affirmatively. The median follow-up was 2 years^70^. In another long-term cohort, 73.7% of patients reported improvement in clinical symptoms, with no complications observed^22^. In the long-term follow-up of patients with pancreatic cancer (PCa), predictors of PERT prescription included a positive EPI test, pancreatic surgery, and evaluation by a gastroenterologist. Over time, these factors also influenced adherence^16^. Retrospective database studies of PCa patients demonstrated that PERT was associated with a statistically significant survival benefit in both resected and unresectable cases, including subgroups with and without chemotherapy^59^ ^,^ ^60^. A prospective, controlled study compared pharmacological presentations of standard-strength versus high-dose capsules. No differences were observed in daily fecal fat excretion, stool volume, or BMI evolution between groups. At study completion, 36% of patients preferred standard-dose pancreatin, while 22% preferred high-dose pancreatin^51^. The occurrence of maldigestion and malnutrition was also investigated in 14 patients who underwent pancreaticoduodenectomy with Wirsung’s duct occlusion using neoprene. Before hospital discharge, mean fecal fat excretion was 32-39 g/day without enzyme replacement, decreasing to 14.2 g/day with PERT. Patients were discharged on a low-fat diet (50 g/day). Remarkably, 6 months postoperatively, mean fecal fat excretion further decreased to 8.3 g/day (p<0.01), and all but one patient gained weight, achieving 93% of their usual mean body weight. These findings indicate that the combination of PERT and a low-fat diet enables effective correction of steatorrhea and significant improvement in nutritional status^5^.

## CONCLUSIONS

Pancreatic and upper gastrointestinal surgeries are significant risk factors for de novo EPI or for the worsening of pre-existing EPI. Subclinical manifestations, nonspecific symptoms, and limited awareness of the condition may delay diagnosis, resulting in nutritional impairment and reduced quality-of-life. The role of this unprecedented Brazilian consensus, validated by national surgical societies, is to provide a foundation for the development of refresher and training programs for surgeons.

## Data Availability

The information regarding the investigation, methodology, and data analysis of the article is archived under the responsibility of the authors.
